# Quick efficacy seeking trial (QuEST1): a novel combination immunotherapy study designed for rapid clinical signal assessment metastatic castration-resistant prostate cancer

**DOI:** 10.1186/s40425-018-0409-8

**Published:** 2018-09-18

**Authors:** Jason M. Redman, Seth M. Steinberg, James L. Gulley

**Affiliations:** 1 Genitourinary Malignancies Branch, Center for Cancer Research, National Cancer Institute, National Institutes of Health, 10 Center Dr., Room B2L312, Bethesda, MD 20892 USA; 2 Biostatistics and Data Management Section, Office of the Clinical Director, Center for Cancer Research, National Cancer Institute, National Institutes of Health, 10 Center Dr., Bethesda, MD 20892 USA; 3 Biostatistics and Data Management Section, Office of the Clinical Director, Center for Cancer Research, National Cancer Institute, National Institutes of Health, 9609 Medical Center Drive, Room 2W334, MSC 9716, Bethesda, MD 20892 USA

**Keywords:** Combination immunotherapy, Metastatic castration-resistant prostate cancer, IDO1, IL-15, PD-L1, Brachyury, Tumor vaccine, TGF-β, ALT-803, M7824

## Abstract

Advances in immunotherapy utilizing immune checkpoint inhibitors (ICIs) have transformed the treatment landscapes of several malignancies in recent years. Oncologists are now tasked with extending these benefits to a greater number of patients and tumor types. Metastatic castration-resistant prostate cancer (mCRPC) infrequently responds to ICIs, while the cellular vaccine approved for mCRPC, sipuleucel-T, provides a 4-month survival benefit but does not produce clinical responses as monotherapy. However, many novel and generally well-tolerated immune oncology agents with potential for immune synergy and/or additive effects are undergoing clinical development. This availability presents opportunities to develop adaptive-design combination clinical trials aimed to generate, expand, and facilitate antitumor immune responses. Here we describe a currently accruing phase I/II trial (NCT03493945) testing a brachyury-targeted antitumor vaccine, TGF-β TRAP/anti-PD-L1 antibody, an IL-15 agonist, and an IDO1 inhibitor in mCRPC.

**Trial registration:** This trial (NCT03493945) was registered in National Clinical Trials on April 11th 2018.

## Background

Immune checkpoint inhibitors (ICIs) have produced dramatic and durable responses for some cancer patients during the past decade [1–7]. Unfortunately, less robust ICI efficacy has also been observed in some malignancies, including prostate cancer [8, 9].

A proposed explanation for these observations is that ICIs are not useful for treating “cold” tumors, i.e., tumors lacking a phenotype characterized by immune inflammation and an underlying recognition of tumor by the immune system. However, recent analyses of over 10,000 tumor samples of varying tumor types identified 6 immune signatures associated with prognosis [10], implying that referring to tumors as “hot” or “cold” is an oversimplification. Nonetheless, metastatic castration-resistant prostate cancer (mCRPC) typically lacks immune infiltrate, and response rates to ICIs have been modest at best [11–14]. In light of these observations and those in various murine tumor models suggesting that combination therapies that generate, expand, and facilitate the function of immune effector cells can lead to enhanced antitumor activity, combination approaches appear to be a promising strategy for potentially enhancing ICIs’ activity in mCRPC and other malignancies.

**Fig. 1:**
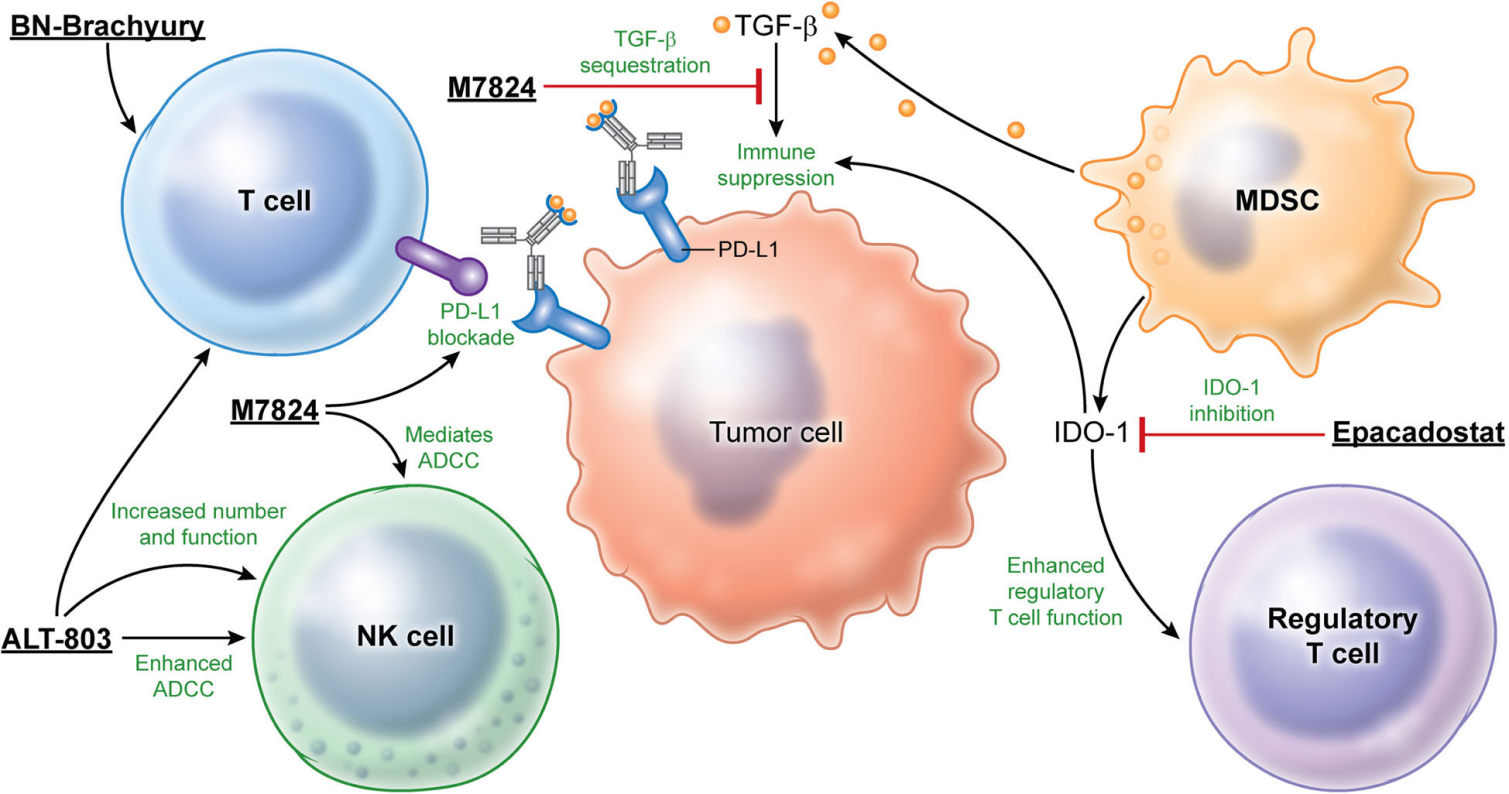
Multimodal immunotherapy can engage, expand, and enable the antitumor immune response. BN-Brachyury vaccine generates T-cell responses by targeting brachyury, a transcription factor involved in metastasis and associated with mCRPC aggressiveness. PD-L1 blockade by M7824 at the tumor:effector cell synapse can enhance tumor lysis. TGF-β neutralization by M7824 can further enable immune effector cell activity within the TME. ALT-803 expands and activates NK cells and effector T cells. Inhibition of the IDO1 enzyme by epacadostat can decrease immunosuppressive currents within the TME generated by myeloid-derived suppressor cells (MDSCs) and regulatory T cells toward a more immune-permissive state within the TME by dampening the inhibitory effects of MDSCs

With many promising novel agents emerging from the clinical pipeline, a disadvantage of traditional approaches to clinical trial design is the extended period of time required to investigate safety and efficacy. Novel trial designs that evaluate multiple agents within one trial offer a means to expedite investigation of multimodal immunotherapy regimens. Based on the rationale outlined below, 4 agents with potential for immune synergy and/or additive effects (Fig. 1) were selected for combination in this adaptive-design clinical trial. Patients will receive one of 4 possible combinations (Table 1).

**Table 1:** Treatment arms

Treatment Regimen for Any Solid Tumor	
Arm 1.1 M7824 + ALT-803	
Treatment Regimen for mCRPC	
Arm 2.1: BN-Brachyury + M7824	
Arm 2.2: BN-Brachyury + M7824 + ALT-803	
Arm 2.3: BN-Brachyury + M7824 + ALT-803 + epacadostat	

### Rationale for combination agents in mCRPC

Sipuleucel-T, a prostatic acid phosphatase-targeted cellular vaccine product, is approved by the U.S. Food and Drug Administration for minimally symptomatic/asymptomatic mCRPC. It is the only immunotherapy currently approved for prostate cancer. This approval was based on phase III testing that demonstrated an overall survival benefit of 4.1 months compared to placebo. Lack of objective responses leaves much room for improvement [15]. A recently published analysis of 57 mCRPC samples found 31.6% to be positive for programmed death ligand 1 (PD-L1) by immunohistochemistry [16]. Since PD-L1 upregulation is a means of immune escape exploited by tumors facing immune attack, lack of immune infiltrate is a potential explanation for why programmed cell death protein 1 (PD-1) and PD-L1 blockade appears not to be relevant in most cases of mCRPC. A study in the neoadjuvant setting demonstrated that the sipuleucel-T vaccine can increase immune infiltrate in tumor. In men who received sipuleucel-T prior to radical prostatectomy, 57% (95% CI: 39–79) had a 3-fold increase in activated T cells in resected prostate samples [17]. This effect was not observed in controls and provided proof of concept that a tumor-targeted vaccine can increase prostate tumor immune infiltrate, potentially creating an environment in which PD-1/PD-L1 blockade may be useful. This suggests that inclusion of tumor-targeted vaccines is important in combination immunotherapy approaches.

BN-Brachyury is a novel recombinant vector-based therapeutic cancer vaccine targeting brachyury, a transcription factor that plays a key role in epithelial-mesenchymal transition (EMT), a critical process for metastasis and drug resistance [18]. In prostate cancer, brachyury expression is associated with aggressive disease [19]. The BN-Brachyury vaccine regimen incorporates the triad of human T-cell costimulatory molecules (TRICOM) platform, encoding B7.1, LFA3, and ICAM-1. Dosing consists of 2 Modified Vaccinia Ankara (MVA)-derived priming doses (MVA-BN-Brachyury), followed by fowlpox-derived booster doses (FPV-Brachyury). Phase I testing showed MVA-brachyury to be well tolerated and capable of generating brachyury-specific T-cell responses at all dose levels [20].

M7824 (MSB0011359C) is an innovative first-in-class bifunctional fusion protein composed of a human IgG1 monoclonal antibody against PD-L1 fused with 2 extracellular domains of transforming growth factor beta (TGF-β) receptor to function as a TGF-β “trap.” In addition to blocking PD-L1 interactions and sequestering TGF-β, M7824 also mediates antibody-dependent cellular cytotoxicity (ADCC) in vitro [21]. Preclinical studies have shown that M7824 may be capable of reversing EMT and increasing response to chemotherapy [22]. Moreover, the potency and magnitude of antitumor responses in murine models appear to be greater with M7824 treatment than with anti-PD-L1 or anti-TGF-β monotherapy [21]. Recent studies performed on tumor samples from metastatic urothelial carcinoma patients demonstrated that half of patients’ tumor samples exhibited a T-cell exclusion phenotype. The investigators observed an association between a TGF-β expression signature in these tumors and responsiveness to atezolizumab (anti-PD-L1) in these patients. The investigators went on to demonstrate that, in a murine model, this T-cell exclusion can be reversed with an anti-PD-L1 and anti-TGF-β treatment combination [23]. In a phase I trial in solid tumors (NCT02699515), M7824 showed evidence of clinical activity (prolonged objective responses) and had a manageable safety profile [24]. A report from an expansion cohort in heavily pretreated recurrent or refractory gastric/gastroesophageal junction adenocarcinoma also demonstrated promising clinical activity. Partial responses were observed in 5/31 patients (16.1%), 4 of which were durable after a 4- to 6-month follow-up period [25]. Taken together, these preliminary observations suggest combined neutralization of PD-L1 and TGF-β effects within the tumor microenvironment (TME) is promising, including after administration of tumor-targeted vaccine.

ALT-803 is an interleukin (IL)-15 superagonist/IL-15 receptor α (IL-15R) fusion complex that can enhance the number and function of both natural killer (NK) cells and effector T cells via agonism of the IL-2 and IL-15βγ receptors, which may lead to enhancement of ADCC and synergy with M7824 [26–28]. ALT-803 is able to reverse an inactivated phenotype and rescue lytic activity of NK cells exposed to TGF-β in vitro [29]. Although ALT-803 and M7824 have never been tested in combination, recently published results from a phase I dose-escalation trial of ALT-803 plus nivolumab (anti-PD-1) in non-small cell lung cancer (NSCLC) demonstrated a favorable safety profile and clinical activity, including activity in ICI-refractory NSCLC [30].

Indoleamine 2,3-dioxygenase-1 (IDO1), an enzyme overexpressed in many solid tumors, catalyzes the conversion of tryptophan to N-formyl-kynurenine (kynurenine). Production of kynurenine and other metabolites by IDO1 can cause T-cell G1 arrest, T-cell and dendritic-cell apoptosis, dampening of NK-cell activity, and enhanced regulatory T-cell activity [31–34]. Epacadostat is a selective inhibitor of IDO1 under investigation in several malignancies. In murine tumor models, epacadostat enhanced the antitumor activity of ICIs [35] and has been shown to have activating effects on the immune system in ex vivo human studies [36]. Although data from a phase I/II dose-escalation trial combining epacadostat with nivolumab 3 mg/kg every 2 weeks indicate that anti-PD-1 combined with IDO1 inhibition has antitumor activity and an acceptable toxicity profile in humans [37], it was announced in April 2018 that the phase III ECHO-301/KEYNOTE-252 study of pembrolizumab (anti-PD-1) plus epacadostat in advanced melanoma did not meet its first primary endpoint of progression-free survival (PFS) (HR 1.00). It is unclear if this result is due to epacadostat inactivity or lack of additional benefit in this particular combination. Nonetheless, IDO1 inhibition may still be important in the context of effects derived from antitumor vaccine, PD-L1 blockade, TGF-β sequestration, and/or tumor inflammation generated by ALT-803.

Some data suggest sustained PSA decline > 30% sustained for > 21 day is useful as a surrogate endpoint for overall survival in patients receiving 2nd line chemotherapy for mCRPC [38]. Since QuEST1 aims to quickly identify signals of activity with the use of novel immunotherapy combinations, in addition to objective response, PSA decline > 30% sustained for > 21 days is used to evaluate efficacy. In the absence of agents that cause fluctuations in the androgen milieu (e.g. enzalutamide or abiraterone acetate), sustained PSA decline is likely to represent anti-tumor activity.

## Methods

### Patients

QuEST1 enrolls patients ≥18 years old with an Eastern Cooperative Oncology Group performance status of ≤1, normal organ and bone marrow function, and histologically or cytologically proven prostate cancer that is castration-resistant, i.e., testosterone levels < 50 ng/dL or 1.7 nmol/L despite androgen-deprivation therapy (ADT). Patients enrolling in arms 2.1, 2.2, and 2.3 must have radiographically proven metastases or prostate-specific antigen (PSA) progression defined as rising values separated by > 1 week, i.e., 2 separate increasing values over a minimum of 1 ng/mL (Prostate Cancer Working Group 3 PSA eligibility criteria). Arm 1.1 is open to patients with any metastatic solid tumor. mCRPC patients will continue on ADT (or are status post-bilateral orchiectomy) and must be minimally symptomatic/asymptomatic and not require regular use of narcotic analgesics. Patients on chronic immunosuppression within 28 days of enrollment, positive for human immunodeficiency virus, or with active autoimmune disease are excluded. Patients with type 1 diabetes mellitus, vitiligo, psoriasis, hypo- or hyperthyroid disease not requiring concurrent immunosuppression, or with other endocrine disorders on replacement hormones are not excluded if the condition is well controlled. mCRPC patients with a history of brain/leptomeningeal metastases are excluded. Eligible patients must also be ≥28 days post major surgery and receipt of other investigative or chemotherapeutic oncologic agents or radiation treatment (with the exception of bone-directed palliative radiotherapy). Concurrent use of agents that can decrease PSA (e.g., saw palmetto) is prohibited in mCRPC patients.

### Study design

**Fig. 2:**
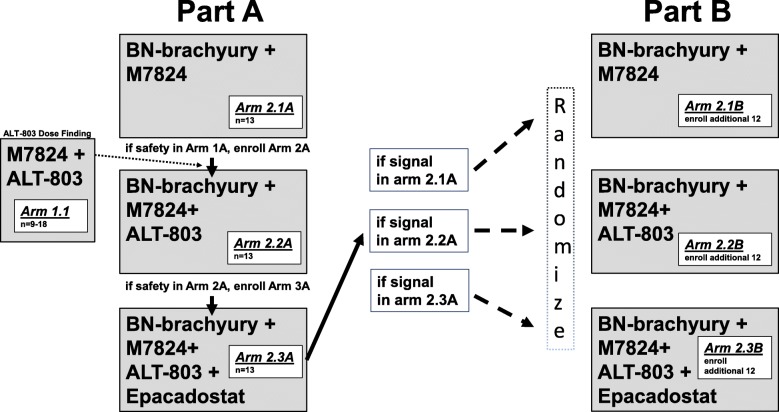
Trial schema. During part A, enrollment to arms 1.1 and 2.1A begins simultaneously. Arm 1.1 is a dose-finding arm for ALT-803 in combination with M7824, open to all solid tumors. After arm 2.1A completes accrual and safety of the combination has been demonstrated, and ALT-803 dosing has been determined from arm 1.1, arm 2.2A begins accrual. After arm 2.2A completes accrual and safety of the combination has been demonstrated, enrollment to arm 2.3A begins. Each of the 3 arms enrolls a total of 13 patients during part A. At completion of part A, if there is a positive safety signal and a positive efficacy signal in arm 2.1A, 2.2A, or 2.3A, part B will begin. To further assess efficacy, arms in which an activity signal was observed (arms 2.1B, 2.2B, and/or 2.3B) may expand to a total of 25 patients. During part B, patients are randomized among all open arms to avoid selection bias

QuEST1 features an adaptive 2-part trial design (Fig. 2). In part A, accrual to each arm occurs sequentially, with each sequential arm adding an immunotherapy agent. Part A also includes an arm (1.1) open to any solid tumor type that will assess maximum tolerated dose (MTD) of ALT-803 in combination with M7824. In part B, immunotherapy combinations that demonstrated safety and preliminary efficacy signals in mCRPC patients will expand. If a patient is removed from treatment, that patient will not be allowed to enroll on another study arm.

### Drug administration

Treatment is given in 2-week cycles (Table 2). Patients receive the priming dose of MVA-BN-Brachyury 2.0 × 10^8^ nfectious units subcutaneously (s.c.) every 2 weeks for 2 doses. Boost doses of FPV-Brachyury 1 × 10^9^ infectious units s.c. begin 2 weeks following the second priming dose and continue every 2 weeks for 6 doses, followed by every 3 months for 2 years from the time of enrollment. In order to assess the MTD of ALT-803 in combination with M7824, a cohort of patients with any solid tumor (*n* = 9–18) will enroll in parallel to arm 2.1A. M7824 will be given at a fixed dose of 1200 mg intravenously (i.v.) every 2 weeks, with dose-level adjustments for s.c. ALT-803 administration (Table 3). An MTD finding of ALT-803 in combination with M7824 must be completed prior to accrual to arm 2.2A. Patients receiving epacadostat (arms 2.3A and 2.3B, if opened) will receive epacadostat 100 mg twice daily by mouth.

**Table 2:** Immune oncologic agents and dosing

Drug	Manufacturer	Dose
BN-MVA-Brachyury (prime doses)	Bavarian Nordic	2.0 × 10^8^ infectious units s.c. every 2 weeks for 2 doses
BN-Brachyury-FPV (boost doses)	Bavarian Nordic	1 × 10^9^ infectious units s.c. begin 2 weeks following the 2nd priming dose, continued every 2 weeks for 6 doses, then every 3 months for 2 years
M7824	EMD Serono, Inc.	1200 mg i.v. every 2 weeks
ALT-803	Altor BioScience	Given s.c. every 2 weeks (see Table 2)
Epacadostat	Incyte, Corp.	100 mg by mouth twice daily

**Table 3:** MTD finding of ALT-803 in combination with M7824

Dose-Escalation Schedule		
Dose Level	ALT-803	M7824
Level − 1	8 mcg/kg s.c.	1200 mg i.v.
Level 1	10 mcg/kg s.c.	1200 mg i.v.
Level 2	15 mcg/kg s.c.	1200 mg i.v.

### Dose-limiting toxicity

Dose-limiting toxicity (DLT) criteria are based on the National Cancer Institute’s Common Terminology Criteria for Adverse Events (CTCAE) version 5. A DLT is defined as any one of the following adverse events (possibly attributable to study drugs) that occur within 21 days of the start of therapy: Any grade ≥ 4 hematologic toxicity or grade 3 thrombocytopenia (platelets 25–50,000) with associated bleeding, except CD4 lymphocyte count or other T lymphocyte subset count. Any grade ≥ 3 nonhematologic toxicity, with the exception of transient (≤ 48 h) grade 3 fatigue, local reactions, flu-like symptoms, fever, headache, nausea, emesis, and diarrhea not controlled with adequate medical management, any CTCAE grade 3 skin toxicity lasting < 5 days, or asymptomatic grade 3 lipase or amylase elevation. Since the goal of the safety evaluation for the combination is to determine if there are any increased or unexpected toxicities due to the combination of therapies that would not be expected with either agent alone, observation of a grade ≥ 3 toxicity known to be associated with either of the 4 investigational agents would not be considered a DLT.

### Safety assessments

The first patient on each arm must be observed for DLTs for at least 13 days. If no DLT is observed within that period, a second patient can enroll and begin treatment. An interval of 2 days without a DLT must pass before treatment of the next patient until after patient 7 begins treatment i.e. before treating patients 3, 4, 5 and 6. A treatment combination will be determined safe if < 2 of the first 6 patients enrolled in a treatment arm experience a DLT.

### Response

The primary endpoint of this study is efficacy, defined as a sustained (> 21 days) PSA decline of ≥30% and/or an objective response in a measurable lesion as defined by RECIST version 1.1.

### Correlates

Peripheral blood samples will be collected on day 1 of cycles 1, 2, 6, and 12. Analyses of peripheral blood include number and function of circulating antigen-specific T cells, levels of sCD40L, sCD27, serum cytokines and chemokines, and circulating immune cell phenotype. When possible, patients will undergo optional biopsies for further analysis.

### Statistical considerations

The primary objective of this trial is to determine if there is clinical benefit, defined as objective response or PSA decline of ≥30% sustained for 21 days, with any of a set of 3 combination treatments (Table 1) for patients with mCRPC. Phase II data from a trial of PSA-TRICOM vs. placebo in mCRPC demonstrated an objective response rate of 0% in measurable disease (in placebo and controls) and a PSA decline of ≥30% in 0% of patients receiving placebo [39]. This historic information is the basis for the statistical plan described below.

### Statistical plan

This study seeks to establish the potential utility of each of the regimens employed. The primary objective is to determine if use of a combination regimen rules out a 10% efficacy rate and results in an efficacy rate consistent with 35%. As such, each arm of the trial is conducted using a Simon minimax 2-stage phase II trial design [40] in order to rule out an unacceptably low partial response + complete response or PSA decline of > 30% (“efficacy”) rate of 10% (*p*0 = 0.10) in favor of an improved efficacy rate of 35% (*p*1 = 0.35). There is no multiplicity adjustment for the 3 Simon 2-stage designs. With α = 0.05 (probability of accepting a poor treatment = 0.05) and β = 0.10 (probability of rejecting a good treatment = 0.10), the first stage for each arm will enroll 13 evaluable patients. If 0–1/13 demonstrate efficacy, then no further patients will be accrued in that arm. If ≥2 of the first 13 patients demonstrate efficacy, then that arm will accrue an additional 12 patients to a total of 25 evaluable patients once stage II begins.

After accruing all 39 evaluable patients in part A, part B will begin. In part B, patients will be randomized among all arms qualifying for expansion as described above. If more than one expansion arm (2.1B, 2.2B, and/or 2.3B) opens, patients will be randomized among those arms. Which arms are available at a given time will depend on whether the efficacy results are known and sufficiently promising for them to continue to enroll patients into part II. The randomization will continue for all included arms until 25 evaluable patients are enrolled in each arm. This may result in some patients never being randomized, but rather accrued to the single open available arm if only one arm is available to accrue patients. Thus, randomization will take place for part B patients during the time in which ≥2 arms are open for accrual; otherwise, accrual will take place without randomization to the one open arm. By this algorithm, there is no bias in assignment to treatment, and the aim is to randomize as many patients as practicable while any others are enrolled directly into the only available arm.

Following an arm’s expansion to 25 patients, 2–5/25 patients with efficacy would be an uninterestingly low rate. If ≥6/25 (24.0%) experience efficacy it would be considered sufficiently interesting to warrant further study. Under the null hypothesis (10% efficacy rate), the probability of early termination is 62.1%. At the end of the trial, the arms will be evaluated with respect to safety and clinical benefit/efficacy. The fraction of patients who experience treatment efficacy will be reported along with 2-sided 80% and 95% confidence intervals. In the absence of appreciably worse toxicity, the arm with the greatest fraction and number of patients experiencing efficacy will be considered for evaluation in future studies. Baseline demographic data on all patients in the phase II cohort will be reported separately by arm.

Each of the 3 phase II arms in the phase II cohort (sequential cohorts) will use a modified intention-to-treat population. Only those patients who have measurable disease present at baseline (including a PSA determination), have received ≥1 cycles of therapy, and have had their disease re-evaluated will be considered evaluable for response. (Note: Patients who exhibit objective disease progression prior to the end of cycle 1 will also be considered evaluable.)

The secondary objectives of this trial are to determine PFS and correlate immunologic measures with clinical outcomes. PFS will be estimated from the on-study date until progression or death without progression. The 6-month PFS probability will be estimated and reported with a 95% confidence interval. Correlation of immunologic outcomes with clinical data and outcomes will be performed by testing the association between the immune outcomes and clinical outcomes, using appropriate nonparametric techniques, such as comparing those with and without efficacy (clinical benefit) with a Wilcoxon rank sum test separately within each arm.

## Conclusion

The responses observed in patients treated with ICIs represent some of the most striking advances in immunotherapy during the past decade. Unfortunately, with rare exceptions, ICI monotherapies are largely inert in mCRPC patients.

Preclinical and clinical studies suggest that the immune effects of tumor-directed vaccine, PD-L1 blockade, TGF-β sequestration, IL-15 agonism, and IDO1 inhibition can be additive and/or synergistic. These include TGF-β’s putative role in T-cell exclusion from the TME in metastatic urothelial carcinoma that can be reversed with dual TGF-β and PD-L1 targeting [23]. Across tumor types, this may create a situation in which activation and expansion of vaccine-derived T cells by ALT-803 may be especially relevant with IDO1 inhibition’s dampening of the inhibitory milieu within the TME.

Here we present an open and accruing adaptive-design clinical trial of combination immunotherapy in mCRPC. This quick efficacy-seeking trial (QuEST1) will expedite testing of these combinations’ ability to generate, expand, and facilitate antitumor activity by measuring objective responses and sustained PSA decline. This novel “fail early or win early” strategy can identify inactive combinations early in the treatment process and allow for immediate interrogation of the next combination.

## Abbreviations

ADCC: Antibody-dependent cellular cytotoxicity
ADT: Androgen-deprivation therapy
CTCAE: Common Terminology Criteria for Adverse Events
DLT: Dose-limiting toxicity
EMT: Epithelial-mesenchymal transition
i.v.: Intravenously
ICI: Immune checkpoint inhibitor
IDO1: Indoleamine 2,3-dioxygenase-1
IL: Interleukin
mCRPC: Metastatic castration-resistant prostate cancer
MDSC: Myeloid-derived suppressor cell
MTD: Maximum tolerated dose
MVA: Modified vaccinia Ankara
NK: Natural killer
NSCLC: Non-small cell lung cancer
PD-1: Programmed cell death protein 1
PD-L1: Programmed death ligand 1
PFS: Progression-free survival
PSA: Prostate-specific antigen
s.c.: Subcutaneously
TGF-β: Transforming growth factor beta
TME: Tumor microenvironment
TRICOM: Triad of costimulatory molecules
